# Diffusion and reactivity in ultraviscous aerosol and the correlation with particle viscosity†
†Electronic supplementary information (ESI) available. See DOI: 10.1039/c5sc03223g


**DOI:** 10.1039/c5sc03223g

**Published:** 2015-11-10

**Authors:** Frances H. Marshall, Rachael E. H. Miles, Young-Chul Song, Peter B. Ohm, Rory M. Power, Jonathan P. Reid, Cari S. Dutcher

**Affiliations:** a School of Chemistry , University of Bristol , Bristol , BS8 1TS , UK . Email: j.p.reid@bristol.ac.uk; b Department of Mechanical Engineering , University of Minnesota , 111 Church Street SE , Minneapolis , MN 55455 , USA; c Max Planck Institute of Molecular Cell Biology and Genetics , Dresden , 01307 , Germany

## Abstract

The slow transport of water, organic species and oxidants in viscous aerosol can lead to aerosol existing in transient states that are not solely governed by thermodynamic principles but by the kinetics of gas-particle partitioning. The relationship between molecular diffusion constants and particle viscosity (for example, as reflected in the Stokes–Einstein equation) is frequently considered to provide an approximate guide to relate the kinetics of aerosol transformation with a material property of the aerosol. We report direct studies of both molecular diffusion and viscosity in the aerosol phase for the ternary system water/maleic acid/sucrose, considering the relationship between the hygroscopic response associated with the change in water partitioning, the volatilisation of maleic acid, the ozonolysis kinetics of maleic acid and the particle viscosity. Although water clearly acts as a plasticiser, the addition of minor fractions of other organic moieties can similarly lead to significant changes in the viscosity from that expected for the dominant component forming the organic matrix (sucrose). Here we highlight that the Stokes–Einstein relationship between the diffusion constant of water and the viscosity of the particle may be more than an order of magnitude in error, even at viscosities as low as 1 Pa s. We show that the thermodynamic relationships of hygroscopic response that underpin such kinetic determinations must be accurately known to retrieve accurate values for diffusion constants; such data are often not available. Further, we show that scaling of the diffusion constants of organic molecules of similar size to those forming the matrix with particle viscosity may be well represented by the Stokes–Einstein equation, suppressing the apparent volatility of semi-volatile components. Finally, the variation in uptake coefficients and diffusion constants for oxidants and small weakly interacting molecules may be much less dependent on viscosity than the diffusion constants of more strongly interacting molecules such as water.

## Abstract 2

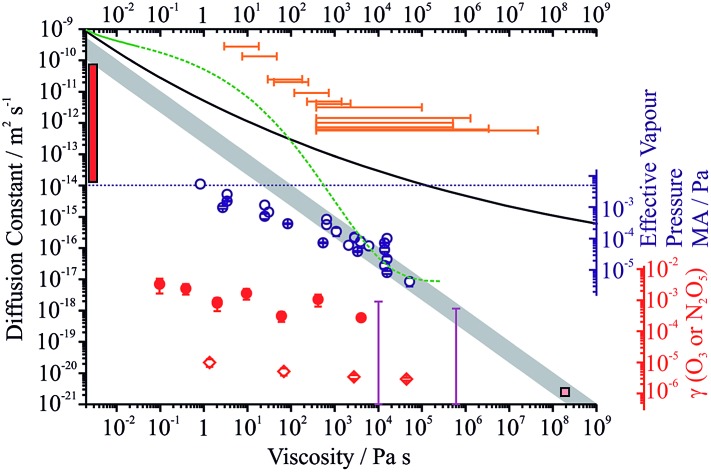
Direct comparison of diffusion coefficients and viscosities of ternary component single aerosol particles levitated using optical tweezers.

## I. Introduction

The chemical composition of aerosol particles is frequently assumed to adjust rapidly to changes in the composition of the surrounding gas phase, maintaining an equilibrium partitioning of volatile and semi-volatile components between the two phases.^1^–^3^ However, it has been demonstrated that slow mass transport within the bulk of viscous, or even glassy, particles may lead to aerosol compositions that are kinetically determined.^4^–^8^ Not only can a low volatility compound require considerable time to volatilise, due simply to the low mass flux into the gas phase, but the slow diffusion of molecules within the bulk of a viscous particle can lead to inhomogeneities in composition and long timescales to achieve equilibrium. In the atmosphere, it has been suggested that the timescale for semi-volatile organic compounds (SVOCs) to achieve an equilibrated partitioning between the gas and condensed phases could be many days depending on the mass loading of aerosol in the atmosphere, even for particles sub-micrometre in diameter.^6^,^8^,^9^ Indeed, not only is it important to quantify diffusivity in viscous aerosol to predict the timescale for SVOC equilibration, the mass loading of organic aerosol and the resulting implications for air quality, but identifying the formation of ultraviscous particles and the inhibition in transport kinetics could be important for understanding the activation of cloud condensation nuclei,^10^–^13^ the activity of ice nuclei^14^,^15^ and the oxidation kinetics of organic aerosol.^16^–^20^

Water acts as a plasticiser with significant changes in viscosity resulting from changes in the extent of particle drying and water content, usually quantified by the water activity or the relative humidity (RH) of the gas phase.^21^ Not only are the material properties of the aerosol sensitive to RH, but the diffusion constants of molecules within the particle bulk can vary by more than 10 orders of magnitude with RH.^22^ The Stokes–Einstein (SE) equation is commonly used to relate the material property, viscosity (*η*), with the molecular diffusion constant (*D*),
1

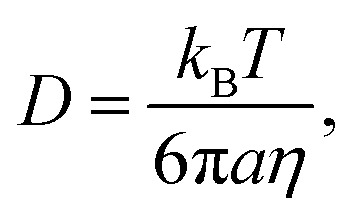

where *a* is the radius of the diffusing molecule, *k*_B_ is the Boltzmann constant and *T* is the temperature. Based on the wide range of viscosities that aerosol particles are expected to access, spanning from dilute aqueous solutions (*η* = 10^–3^ Pa s) through to glassy states (*η* = >10^12^ Pa s),^21^,^23^,^24^ a similarly large range of diffusion constants might be expected. However, the SE equation is known to be inappropriate for estimating diffusion constants for small molecules diffusing through a matrix of large molecules; for example, the diffusion constant for water in a viscous sucrose solution is underestimated by an order of magnitude at the threshold viscosity between a viscous liquid and a semi-solid (∼10^2^ Pa s), with increasing divergence at increasing viscosity.^22^ This should be contrasted with the diffusion constant of sucrose within aqueous sucrose solutions which can be represented accurately by the SE equation to high viscosity.^25^ The inaccuracy in using the SE equation to estimate diffusion constants highlights the need for direct measurements of the kinetics of mass transport of different molecules in viscous aerosol.

We present here a comprehensive study of the correlation between particle viscosity and the kinetics of mass transport of water, a SVOC and ozone in viscous aerosol. The SVOC chosen is maleic acid (MA); not only does MA have a pure component vapour pressure (∼10^–3^ Pa)^26^ that allows evaporation measurements over a convenient timeframe (10^4^ s), but the presence of an olefinic bond provides a clear signature of the progress of oxidation kinetics by Raman spectroscopy. The viscosity of the aerosol is controlled by forming a particle in which sucrose represents the dominant fraction of solute mass; the kinetics of water transport and the dependence of viscosity on RH for sucrose aerosol is well-established.^22^,^27^ Measurements are reported of the kinetics of water and MA evaporation from aqueous MA/sucrose droplets at varying water activity, equivalent to evaporation from particles of differing viscosity, and providing insights into the influence of viscosity on water and SVOC equilibration timescales. The kinetics of the ozonolysis of MA are also investigated at varying water activity, providing insights into the dependence of the heterogeneous reaction rates on particle viscosity. Finally, direct measurements of the viscosity of aqueous MA/sucrose particles are reported at varying water activity, with the objective of examining the correlation between the molecular size and diffusion constant with the viscosity of the particle bulk.

## II. Experimental description

The experimental approach for measuring the ozonolysis kinetics of single aqueous-organic aerosol particles using optical tweezers has been comprehensively described in previous publications and we only briefly summarise the key elements.^28^–^30^ An aqueous droplet containing maleic acid/sucrose is captured from a nebulised cloud of aerosol in a tightly focussed laser beam (wavelength 532 nm) formed through a high numerical aperture oil-immersion objective. The initial mass ratio of the two solutes is known, with subsequent changes occurring over time as MA evaporates. In the experiments described here, the initial mass ratio is 5 : 1 sucrose : MA except where otherwise stated. The RH of the gas phase is controlled from dry conditions (<5% RH) to high RH (>80%) by mixing flows of dry and humidified nitrogen, and the RH and temperature of the gas flow monitored (HUMICAP HMT 330, Vaisala). Particles are imaged by brightfield microscopy. Inelastic backscattered light collected by the microscope objective is imaged into a 0.5 m focal length spectrograph, dispersed by a 1200 g mm^–1^ grating, and the Raman spectrum recorded by a CCD with a time resolution of 1 s and a spectral dispersion of <0.05 nm per pixel. In addition to the familiar spontaneous Stokes bands shifted from the excitation wavelength, the Raman spectrum provides a unique fingerprint of droplet size and refractive index (RI) through the pattern of resonant modes superimposed on the spontaneous band at wavelengths commensurate with whispering gallery modes. The size, RI and dispersion in RI can be retrieved with high accuracy from this fingerprint by comparison with Mie scattering calculations.^31^,^32^ The trapped droplet can also be exposed to ozone produced by an ozone generator (Model 600, Jelight) with the concentration measured by passing the gas flow through a 10 cm long absorption cell, with measurements of absorption made in the UV at a wavelength of 254 nm. The slow ozonolysis kinetics of MA requires concentrations of ozone in excess of 30 ppm for full reaction of MA to proceed on a timescale of 10 000 s. The disappearance of MA and appearance of products can be observed through changes in the spontaneous Raman bands, notably the loss of the vinylic C–H bond stretch between 3025 and 3100 cm^–1^ shift.

Viscosity measurements are also performed using an optical tweezers approach. Instead of capturing a single droplet in a single optical trap, pairs of droplets are captured in two independent traps, formed using holographic optical tweezers. Once a period of time has been allowed to condition the particles at the RH of the trapping cell (typically 500 s), the pair of droplets is coalesced. The time-constant for the relaxation of the composite particle to a sphere can be measured by light scattering or inferred from the brightfield imaging, covering timescales for relaxation of 10^–6^ to 10^–3^ and 10^–3^ to >10 000 s, respectively. The relaxation time can be used to infer the particle viscosity: in a previous publication, we have shown that the viscosity can be inferred over a wide range spanning from 10^–3^ to >10^9^ Pa s.^22^

## III. Evaporation kinetics of water and MA

**Fig. 1:**
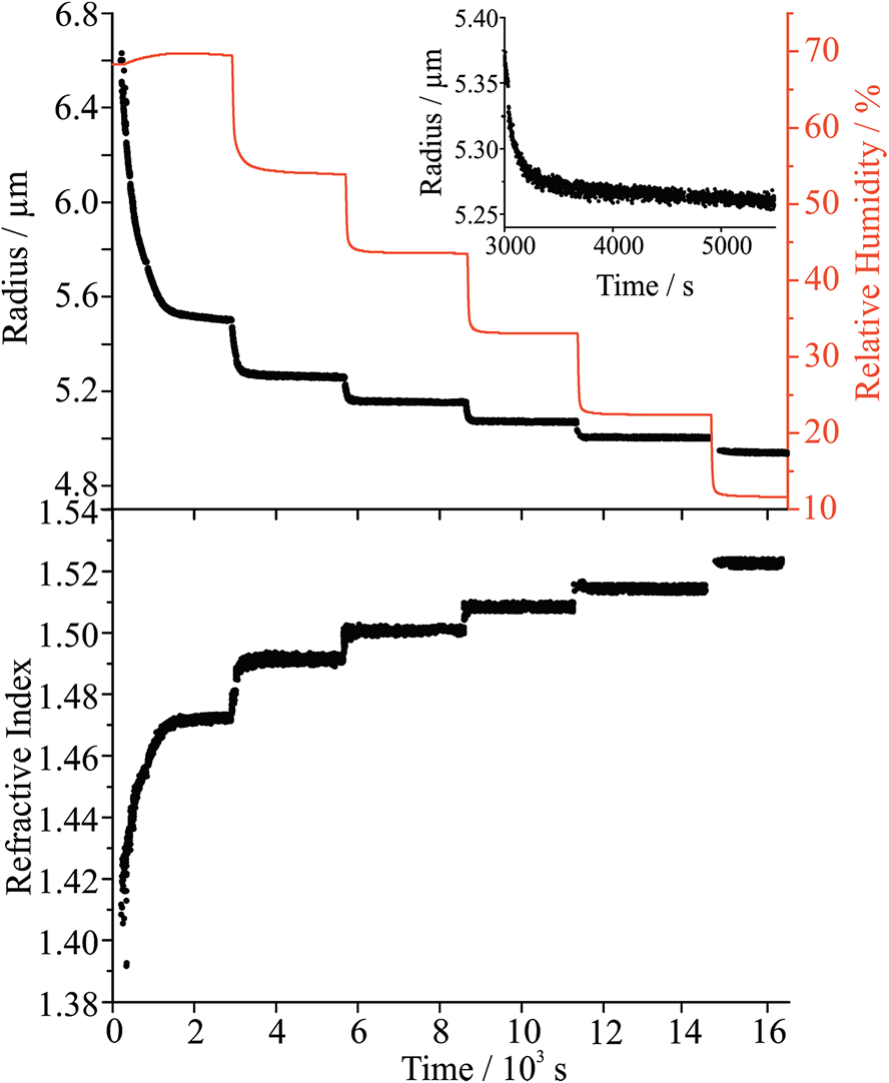
Radius and refractive index of an aqueous MA/sucrose droplet (black points) and RH (red line) during stepwise changes in the RH. The inset shows an expanded view of the steady evaporation of MA at a constant RH of 52%.

To explore the mechanism of water and SVOC evaporation from viscous particles, aqueous droplets of sucrose/MA were optically trapped and the RH varied in a sequence of steps, either by steadily decreasing the RH in small steps (ΔRH ∼ 10%, with an example shown in Fig. 1) or through an immediate and large step (ΔRH > 50%, *e.g.* from 80 to 20%). The wet size of the droplet diminishes through evaporation of water to maintain an equilibrium balance in water activity that matches the decrease in RH; the resulting increase in solute concentration leads to an increase in the RI of the droplet. At each step in RH, the size and RI of the droplet remain responsive, adjusting rapidly to the change in gas phase conditions. In previous studies, we have shown that the typical timescale for a change in the gas phase RH in the instrument is <100 s.^33^

To quantitatively assess the kinetics of water loss from ultraviscous and glassy particles, we have shown that the time-dependence of the droplet size can be described by the Kohlrausch–Williams–Watts (KWW) equation, a stretched exponential response function.^34^,^35^ The temporal dependence of the response function, *F*(*t*), when responding to an applied perturbation is given by:^36^

2 *F*(*t*) ≈ exp[–(*t*/*τ*)^*β*^]

where *τ* is the characteristic relaxation time and *β* (<1) decreases markedly as the system approaches a glass transition. For characterising the water transport in viscous sucrose aerosol we have shown that *β* takes the value 0.5 ± 0.1.^35^ The response function for a change in radius can be expressed as:
3

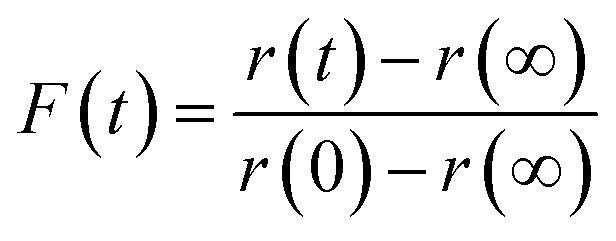

where *r*(*t*) is the evolving time response of the relaxing parameter, here the droplet radius at time *t*, and *r*(0) and *r*(∞) are the initial and final values respectively, *i.e.* the response function is the fractional progression in droplet size from the initial to final states. Alternatively, the response function can be expressed in terms of the RI change.
4

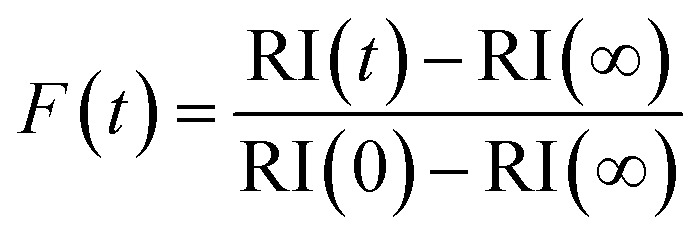




**Fig. 2:**
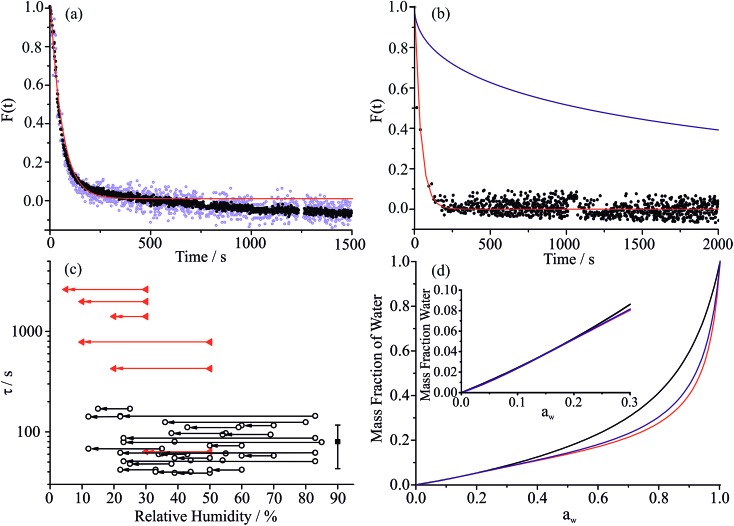
(a) Response functions for radius (black) and RI (purple) for a RH change between 69% and 54%, consistent with a *β* value of 1 (fit, red line). (b) Comparison of the fitted response functions for relaxation in radius for a MA/sucrose droplet (measurements, black circles; fit, red line) and an aqueous sucrose droplet (blue, fit line) experiencing the same RH change (33–23%). (c) Fitted values for the time constant, *τ*, for various RH step changes for MA/sucrose droplets (black circles) and aqueous sucrose droplets (red triangles). The direction of the arrow represents the RH change with the points indicating the initial and final RH between which the RH is changing. The average and standard deviation of the relaxation times for all RH transitions from all MA/sucrose droplets studied is shown arbitrarily at 90% for comparison. (d) Variation in mass fraction of water with water activity for aqueous MA (black), aqueous sucrose (red) and a 5 : 1 sucrose/MA mixture (blue) calculated from the adsorption isotherm model with the inset showing the behaviour at low water activity.

Considering the response functions for size and RI following an RH change, the form of the relaxation is not consistent with a stretched exponential with *β* < 1 for the measurements on maleic acid/sucrose aerosol presented here. Examples of the comparison between the response functions for radius and RI are shown in Fig. 2(a) and (b) for changes in the RH between 69 and 54% and 33 and 23%, respectively. For the transition at higher RH, the initial fast decline in radius and RI response functions can be fit to a single exponential (*β* = 1) followed by an approximately linear decrease in size with time that persists indefinitely due to the slow volatilisation of MA from the particle. The “final” state is taken as the size/RI at the point where the size change becomes linear with time following the initial fast kinetic response for water adjustment to the RH change. For the transition at lower RH (Fig. 2(b)), no persistent loss in size due to MA volatilisation is observed, but the initial loss of water remains fast and can be fit to a single exponential (*β* = 1). For comparison we include a response function measured for a binary aqueous sucrose droplet (*i.e.* no MA) over the same RH step reported from our previous work, illustrating the marked differences in behaviour.^10^,^11^ The time constants for the two response functions are 42 s (aqueous maleic acid/sucrose) and 2274 s (aqueous sucrose) with the time constant for faster water transport in the ternary droplet representative of the timescale of the RH change, rather than slow water transport in the bulk of a viscous particle. Indeed, time constants for water evaporation from sucrose aerosol are considerably larger than from sucrose/MA particles (Fig. 2(c)) when the change in water activity takes the particle below a water activity of ∼0.25 (RH of 25%), the realm of slow water transport in ultraviscous and glassy sucrose aerosol.^10^,^11^

To be resolvable from the instrument response time, the bulk diffusion constant of water must decrease below ∼10^–14^ m^2^ s^–1^. The diffusional mixing time *t*_mix_ (=*r*^2^/(π^2^*D*), where *r* is the droplet radius) is of order 100 s for a 4 μm radius droplet for this value of the diffusion constant; this represents a limit for the diffusion constant above which the water transport kinetics would be unresolved from the timescale for the RH change.^37^ Although a kinetic limitation on water transport was unresolved for drying of a 5 : 1 sucrose : MA particle down to 20% RH (with an average time constant of 80 ± 37 s determined over all measurements, as shown in Fig. 2(c)), a marginal reproducible slowing could be discerned when the RH was decreased below 20% RH. For comparison, aqueous sucrose droplets show a kinetically resolvable water transport limitation with a half time of >100 s when dried to RHs in the range 40–50%, corresponding to diffusion constants in the range 5 × 10^–15^ to 2 × 10^–14^ m^2^ s^–1^.^38^ Given the large uncertainties in estimating the diffusion constant from such a slight degree of resolvable slowing, particularly for a ternary component droplet, we conclude that the diffusion constant must approach ∼10^–14^ m^2^ s^–1^ at 20% RH for 5 : 1 sucrose : MA particles (an order of magnitude assessment only).^23^

The water activity dependencies of the equilibrium compositions of aqueous droplets of sucrose, MA and sucrose/MA (5 : 1 mass ratio) are compared in Fig. 2(d). These dependencies are calculated from the adsorption isotherm model of Dutcher *et al.* based upon a multilayer adsorption isotherm, which accurately represents the compositional dependence of water content to zero solvent activity (the hypothetical pure liquid solute).^39^–^43^ The model expresses solute–water interactions in terms of energies of sorption of the adsorbed solvent into *n* layers surrounding the solute molecule. Using coulombic relationships, the energy of sorption parameters are calculated from intermolecular distances, *d*, and dipole moments, *μ*, for both solvent and solute molecules (eqn (9) in ref. 42). The three adjustable model parameters for the systems studied here (sucrose: *d*_jw_ = 4.55659 Å, *μ*_j_ = 13.923, *n*_j_ = 20; maleic acid: *d*_jw_ = 3.17995 Å, *μ*_j_ = 4.3206, *n* = 3) are found from empirical fits to molality activity data available in the literature. The addition of MA to form a ternary mixture has very little impact on the predicted mass fraction of water in the particle when compared with the composition for a binary sucrose/water mixture. Thus, the faster diffusional kinetics of water in a sucrose/MA particle when compared to a sucrose particle (a factor of more than 2 orders of magnitude at the lowest RH) cannot be attributed to the plasticising effect of water, but must instead reflect the impact that the addition of a minor fraction of MA has on the permeability of water through the particle. This minor fraction must influence the microscopic structure of the particle in such a manner as to make water transport more facile; in short, the MA acts in the same way as a plasticiser on the surrounding sucrose matrix.

**Fig. 3:**
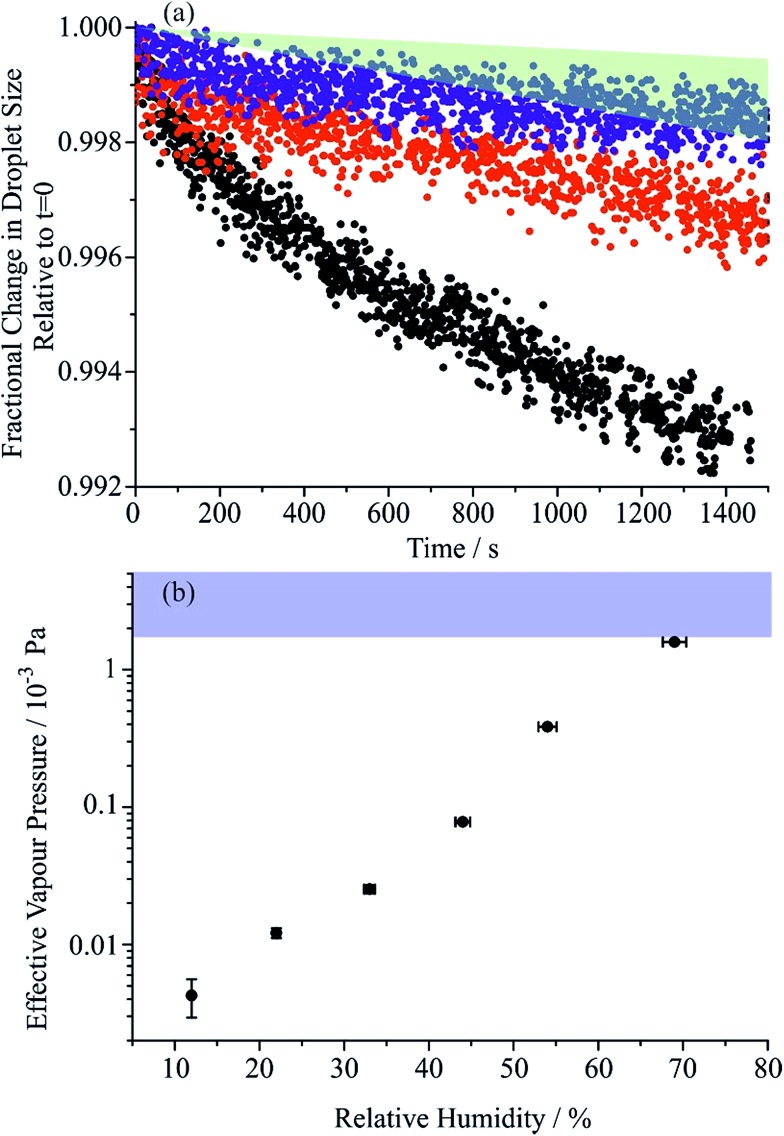
(a) Fractional change in droplet size over 1500 s for a MA/sucrose droplet at three different RHs, 70% (black), 50% (red) and 30% (blue), showing the gradual retardation to the evaporation of MA as the RH decreases. The range of the fractional change for the mixture at 10% RH is shown by the green envelope (data not explicitly shown for clarity). (b) Estimates of the effective vapour pressure at each RH step tend toward the reported pure component vapour pressure of aqueous MA (blue band) at high RHs.

Once the hygroscopic response to the RH change is complete, the subsequent linear decline in particle size arises from the slow volatilisation of the much lower vapour pressure MA component from the droplet, accompanied by the solvating water. The RI and size both continue to evolve with the droplet becoming progressively richer in sucrose as the mass of MA decreases, particularly apparent at higher water activity where MA evaporation is most rapid. The reference values for the pure crystalline and liquid melt RIs of MA are 1.481 and 1.509, respectively, and 1.538 and 1.562 for sucrose. These are consistent with the maleic acid/sucrose droplet RI under dry conditions observed in Fig. 1(b), which falls between the values for MA and sucrose.^44^ In Fig. 3(a) we compare the fractional changes in size that occur following completion of an RH step from a sequence of typical measurements on the same droplet; similar trends, although more noisy, can be observed from the change in composition inferred from the spontaneous Raman band intensity for MA. Contrary to expectations, the gradient in the radius change becomes shallower as the RH is decreased and as the droplet becomes richer in solute. This marked difference cannot be simply attributed to systematic changes in droplet radius with RH or in decreasing MA fraction within the droplet over the course of a measurement. From the relationship of MA vapour pressure above the droplet surface, *p*_MA,*r*_, to pure component vapour pressure, *p*oMA, and droplet composition,

5 *p*_MA,*r*_ = *x*_MA_*f*_MA_*p*oMA,

we would expect the mass flux of MA to increase with a decrease in RH. *x*_MA_ is the mole fraction of MA and *f*_MA_ is the mole fraction activity coefficient relative to the pure liquid reference state.

The effective pure component vapour pressure of MA can be estimated from the rate of change in the radius-squared of an aqueous droplet (*r*^2^) held at a constant RH with time (*t*) due to the evaporation of MA. The Maxwell equation can be written as:
6

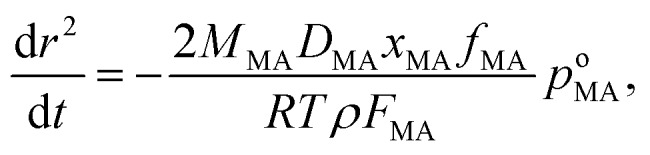

where *M*_MA_ is the molecular mass of MA, *D*_MA_ is the diffusion coefficient of MA in the surrounding gas, *R* is the ideal gas constant, *T* is the temperature, *ρ* is the density of the droplet and *F*_MA_ is the mass fraction of MA in the droplet. The diffusion constant is estimated as 7.2 × 10^–6^ m^2^ s^–1^ in nitrogen at 298.15 K using the equations of Chapman and Enskog and from Lennard-Jones potential parameters.^45^ The partial pressure of MA at infinite distance from the droplet surface (*p*_MA,∞_) is assumed equal to zero due to the continual gas flow around the droplet. Over the timeframe of a single evaporation measurement during a period of constant RH (typically 2000 to 3000 s), we assume that the composition of the droplet is constant.

Estimates of the effective pure component vapour pressures as a function of RH (estimated from eqn (5) and (6)) are shown in Fig. 3(b) with a convergence with the expected value as the RH increases; at the lowest RH the effective vapour pressure of MA is suppressed by more than two orders of magnitude below what is expected and this is a consequence of a kinetic suppression of the evaporation rate. The diffusion constant of MA in aqueous solution is in the range 0.93 to 1.55 × 10^–9^ m^2^ s^–1^, less than the value for water simply because of the molecular size.^46^ However, it is clear that as the sucrose rich particle is dried, a retardation in the diffusion of MA within the particle bulk must depress the diffusion constant well below 10^–14^ m^2^ s^–1^ at water activities at which the diffusion of water remains rapid compared to our experimental timescale. The drying/humidification route taken may influence the homogeneity of the particle at the point at which it is studied, thereby influencing water transport kinetics and MA evaporation. Although we have studied the impact of the particle history on water transport in sucrose aerosol, we do not consider this factor further in this study.^35^

## IV. Reaction kinetics of MA with ozone

**Fig. 4:**
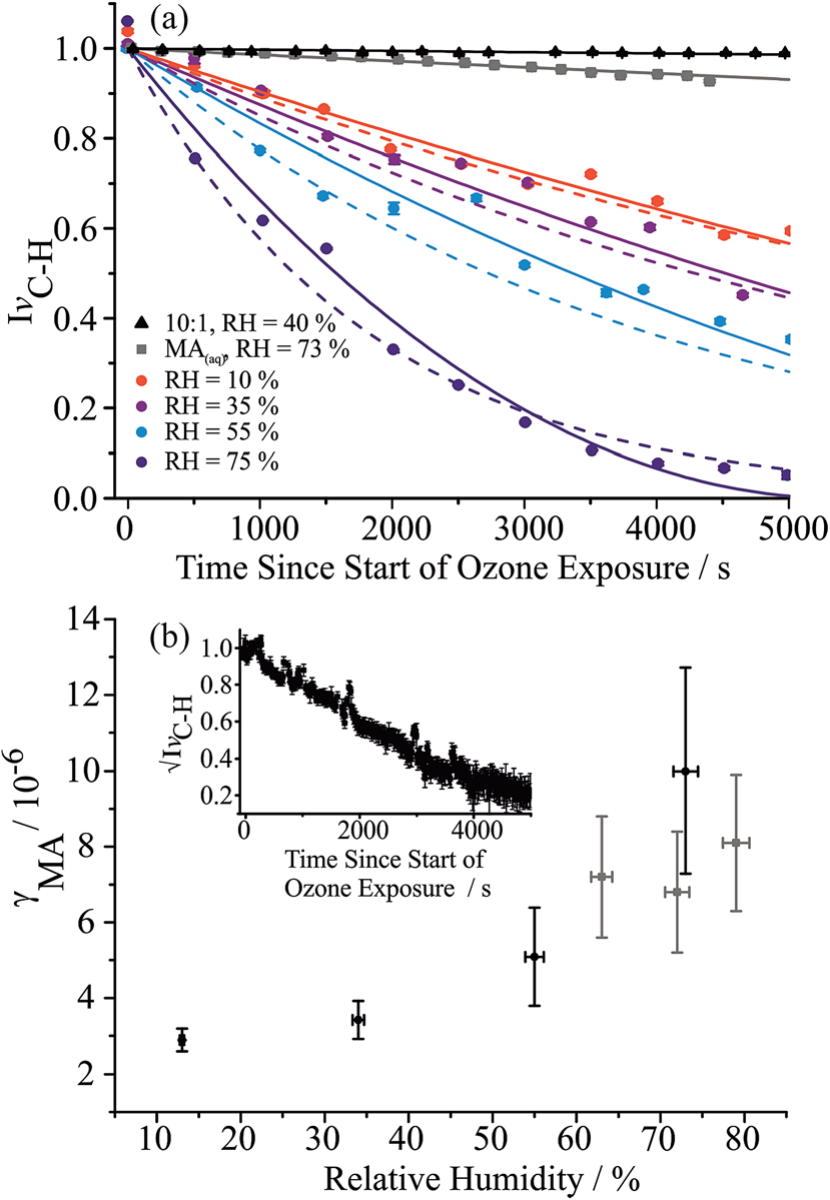
(a) Time-dependence of the normalised spontaneous Raman signal intensity of the MA vinylic C–H stretch during an oxidation experiment at different RHs (75%, 55%, 35%, 10%, dark blue to red circles in sequence) for a 5 : 1 mass ratio of sucrose/MA. Grey squares show change in Raman intensity for an aqueous MA droplet evaporating at 73% without reaction. Black triangles show change in Raman intensity during ozonolysis for a 10 : 1 mass ratio sucrose/MA droplet at 40% RH. Solid and dashed lines are fits assuming that the reaction is bulk diffusion limited or gas-diffusion limited, respectively. (b) RH dependence of the reactive uptake coefficients using the approach of King *et al.* (black circles).^48^ The grey squares are from measurements of the ozonolysis kinetics of an aqueous MA droplet exposed under similar ozone concentrations. The inset shows an example of the time-dependence of the square root of the normalised spontaneous Raman signal intensity.

With a suppression in MA evaporation from a particle at low RH, it might be expected that a suppression in ozonolysis kinetics would be observed due to slow diffusion of ozone into the particle and slow diffusion of MA from the particle core to a near-surface region. Ozonolysis proceeds through fragmentation reactions to form products of lower molecular weight and wide ranging vapour pressure, each of which may be kinetically limited in evaporation:
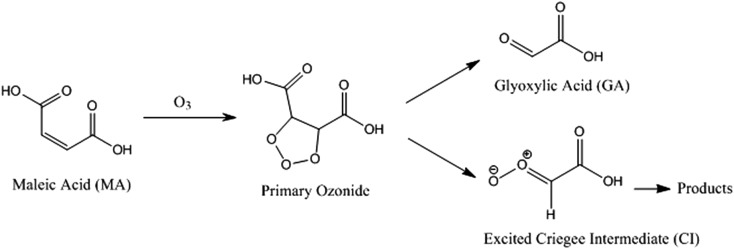


Particles of sucrose/MA were exposed to ozone (typical concentration of ∼36–39 ppm) for timescales of up to 10 000 s at RHs in the range 10 to 75%. During this time, the intensity of the Stokes band characteristic of the vinylic C–H stretch was monitored, providing a direct signature of the rate of reaction of MA and cleavage of the C=C, as shown in Fig. 4(a). In a previous paper we discussed the plausible treatments of the ozonolysis kinetics assuming two limiting cases: a bulk reaction limited by ozone diffusion into the particle followed by reaction, and a gas diffusion limited reaction with near-surface reaction. Both treatments are considered here to explore the robustness of the estimates of the uptake coefficients (see ESI† for further details). Without a kinetic limitation and with a diffusion constant of 1.8 × 10^–5^ cm^2^ s^–1^ in the aqueous phase, the reacto-diffusive length of ozone has been estimated to be between 100 and 270 nm.^47^ These approaches have also been previously compared by King *et al.* to investigate the oxidation of micron-sized aqueous sodium fumarate droplets by ozone.^48^Fig. 4(a) shows fits for both treatments to the variation in MA vinylic C–H Raman intensity with time during ozonolysis of MA/sucrose droplets at varying RH, with uptake coefficients estimated by both methods consistent within ±30%. Within the uncertainties in the measurements, it is not possible to identify which kinetic scheme better reflects the measured values.

From the RH dependence of the extracted uptake coefficients, the suppression in reaction rate observed with decrease in RH is only slight for 5 : 1 sucrose : MA component droplets: even though the suppression in reaction rate is clear from the time-dependent trends, this leads to only a mild change in uptake coefficient with RH. The loss of MA by evaporation also leads to a decrease in the intensity of the vinylic signature and this is shown for comparison in Fig. 4(a), although occurring over a much longer timescale than the reactive loss. The uptake coefficients reported at the highest water activity compare well to the value reported for the reaction of ozone with aqueous droplets of MA, shown in Fig. 4(b). The slight suppression in reactive uptake coefficient for the ternary droplets becomes considerably more marked when the droplet has a sucrose : MA ratio of 10 : 1, decreasing by two orders of magnitude to 3 × 10^–8^. Indeed, this value represents an upper limit as the ozonolysis appears to be completely quenched for particles of this composition, as apparent in Fig. 4(a). The mass fractions of water in particles of 5 : 1 and 10 : 1 mass ratio at 35% and 40% RH, respectively, can be estimated to be 0.096 and 0.110 from the adsorption isotherm model. As observed in the mass transport kinetics of water, the fraction of water does not seem to play the determining role in the value of the reactive uptake coefficient. Instead, the interactions of sucrose and MA in forming the matrix through which small molecules must diffuse (water and ozone) must play the determining role in governing the uptake coefficient.

## V. The water activity dependence of particle viscosity

**Fig. 5:**
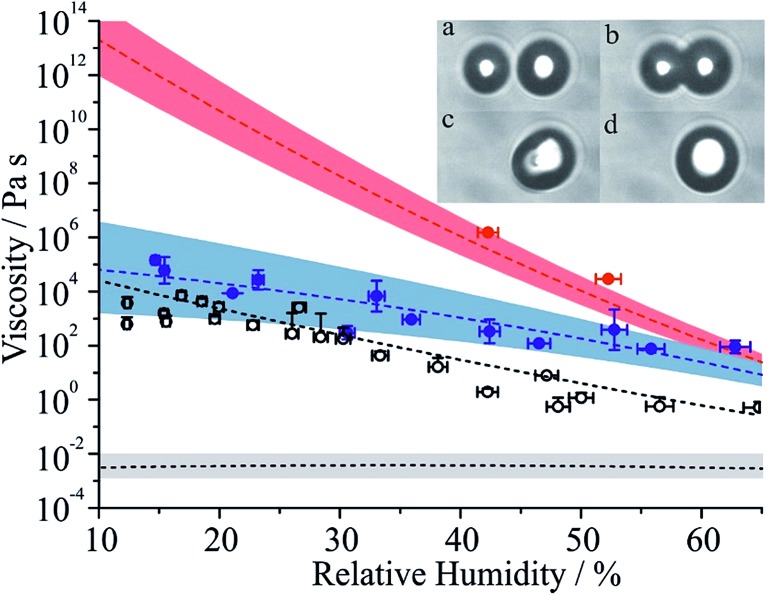
Measured viscosities for MA/sucrose particles as a function of RH with associated fit and error envelope (see Tables S1 and S2 for further information†). Data points are colour coded according to the MA mass fraction of total solute in the final droplet at coalescence (blue > 0.09, red < 0.04). The red dashed line is a fit to the aqueous sucrose data presented in ref. 22, with associated error envelope. The grey envelope (with black dashed line set to a value of 2.5 mPa s) represents the viscosity of an aqueous-MA solution at a mass fraction of 0.40.^49^ Black unfilled circles are measured viscosities for aqueous citric acid droplets with the associated parametrisation shown by the dashed black line. The inset (a–d) shows sequential brightfield images during coalescence of two MA/sucrose particles at an RH of 42% with a time step of 0.015 s between frames.

As suggested by the SE eqn (1), although imperfect, the relationship between diffusion constants and viscosity can provide a guide as to the qualitative trends expected in bulk diffusion and, thus, in aerosol equilibration and reaction timescales. Using the method we have previously described through the coalescence of pairs of droplets and measurements of the timescale for relaxation to a sphere,^22^,^37^ we report direct viscosity measurements for droplets of the sucrose/MA 5 : 1 ratio composition in Fig. 5 (see Table S1 for tabulated values†). The viscosity inferred from the relaxation timescale was determined either from the time-dependence in the elastic light scattering from the merging spheres or from the brightfield imaging, spanning timeframes of 4.9 × 10^–5^ to 4.5 × 10^–3^ s and 0.013 to 515.3 s, respectively. All coalescence events were in the overdamped regime.^22^,^37^ These measurements are compared with the RH dependence for binary sucrose/water droplets and citric acid/water droplets, with the latter system also reported here for the first time in Fig. 5, alongside an example of the sequential brightfield images. The bulk viscosity of a binary MA/water solution in the dilute limit is also shown.^49^

The amount of MA remaining in the particle at the time of each viscosity measurement is estimated from the integration of the MA and sucrose Raman band intensities immediately prior to coalescence. These values suggest that the composition was always less than the 5 : 1 mass ratio intended due to partial volatilisation of MA before the viscosity was measured. Notably some viscosity measurements were made at sufficiently long time after the optical traps were loaded that a considerable fraction of MA had evaporated into the gas phase; for these measurements, the viscosities were found to be very similar to the viscosity of aqueous sucrose droplets. This observation illustrates the challenges involved in making such measurements where the viscosity is exponentially dependent on composition. Despite this, the parameterisations and error envelopes for the water activity dependent fit of viscosities for sucrose/MA (ignoring the points that most closely represent sucrose) and sucrose are given in Table S2.†

Aqueous MA droplets have been shown to effloresce in the range 30–50%.^50^,^51^ As already indicated, kinetic measurements for MA/sucrose particles have been made at RHs as low as 10%, clearly illustrating the suppression in nucleation kinetics and crystallisation that occurs when droplets become viscous and molecular diffusion becomes slow. Although the viscosity of aqueous MA/sucrose does not increase as steeply as aqueous sucrose when the RH is decreased, the viscosity is ∼7 orders of magnitude higher than would be expected for aqueous MA in the absence of sucrose at 20% RH, but remains some 6 orders of magnitude less than aqueous sucrose.^49^ Intriguingly, thermodynamic predictions of compositions of the binary and ternary sucrose/water and sucrose/MA/water droplets at 20% RH suggest very similar mass fractions of water (Fig. 2(d)), reinforcing the conclusion that the presence of water is not the determining factor that governs particle viscosity, but that the interactions of different organic solutes (in this case MA with sucrose) in ternary mixtures can lead to particles of radically different phase behaviour and viscosity. Most measurements of aerosol viscosity and diffusion constants of water have been made on binary solution droplets or on mixtures of complex composition, specifically secondary organic aerosol.

At 20% RH, the viscosity of the MA/sucrose particles can be estimated to be ∼2 × 10^4^ Pa s, equivalent to the viscosity of sucrose/water droplets at 48% RH; as indicated earlier, the water evaporation kinetics from particles of these two compositions are comparable with the diffusion constant of water between 5 × 10^–15^ to 2 × 10^–14^ m^2^ s^–1^ in the range 40–50% RH for the binary system. Particles of similar viscosity but different composition do seem to lead to diffusion constants for water that are similar when the dominant organic component forming the matrix is the same, in this case sucrose.

## VI. Conclusions

The significant suppression in the apparent volatility of MA with diminishing water activity and the reduction in the reactive uptake coefficient of ozone suggest that the bulk diffusion constants of MA and ozone are lowered by the viscosity of the matrix through which they must diffuse. Notably, water transport does not seem to be significantly slowed in ternary aqueous sucrose/MA droplets, with the response times remaining on the timescale for the gas phase change (90 ± 37 s, *D* > 10^–14^ m^2^ s^–1^), even under conditions where the water content (by mass fraction) would show kinetic slowing in the binary sucrose/water system. This observation highlights that it is not simply the plasticising influence of water that regulates the viscosity and diffusion constants in aqueous based organic aerosol, but that the specific functionalities and molecular weights of the organic species forming complex mixtures must be considered. The diffusion constant for water approaches a value of ∼10^–14^ m^2^ s^–1^ at a water activity of 0.2 where the viscosity reaches 2 × 10^4^ Pa s. At this same viscosity, the water activity for binary aqueous-sucrose droplets is ∼0.48 with the timescale for water transport similarly only just resolvable from the instrument response at ∼50% RH (∼100 s).^11^ It should be noted that although Bones *et al.*^11^ reported this resolvable threshold as occurring at a viscosity of ∼10 Pa s (rather than 2 × 10^4^ Pa s), this was based on a parameterisation of aqueous-sucrose viscosities from sub-saturated measurements that was later surpassed in accuracy by direct aerosol measurements.^22^

**Fig. 6:**
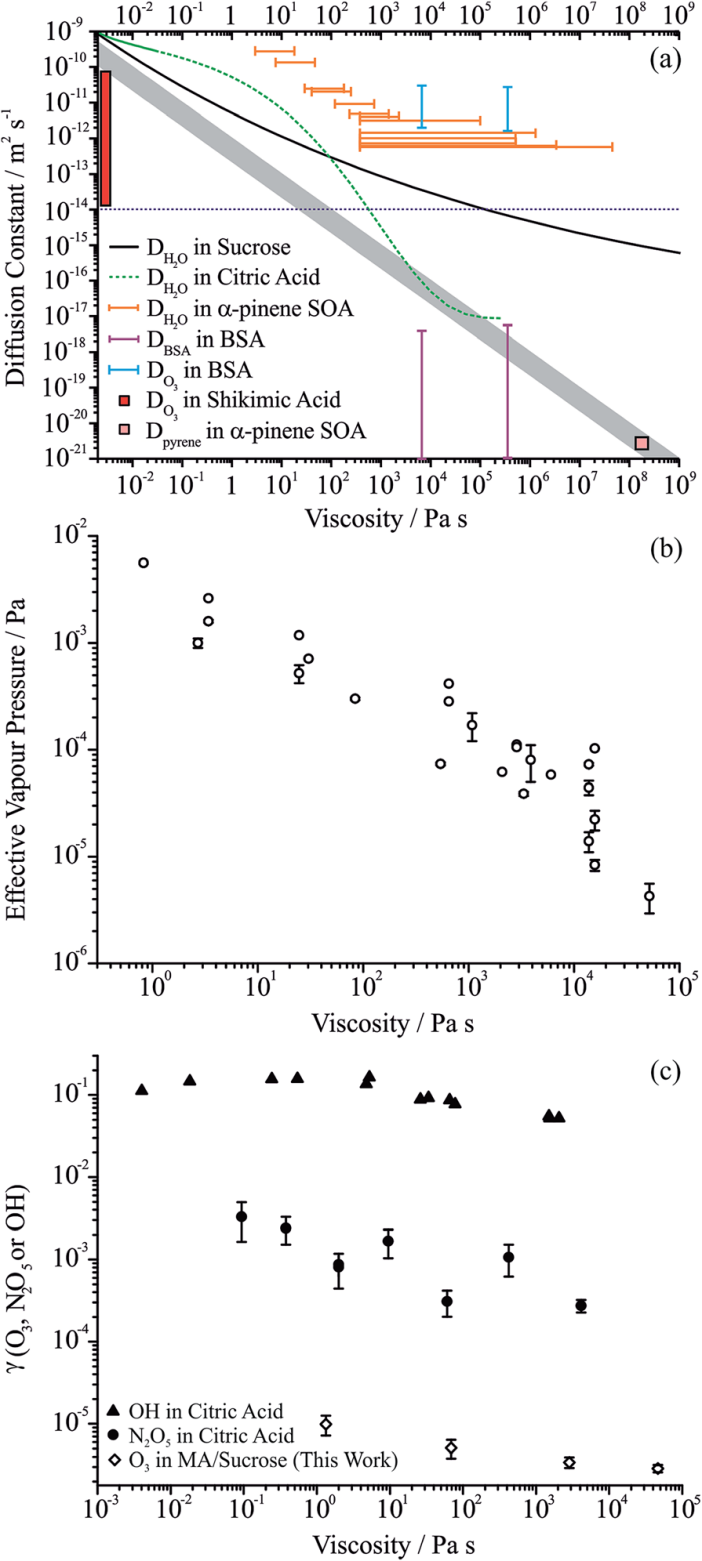
(a) Comparison of the dependence of diffusion constants on viscosity for organic aerosols (see main text for full details). The grey envelope indicates the relationship calculated from the Stokes–Einstein equation with molecular radii of 0.2 and 1 nm (top and bottom of envelope, respectively). Diffusion constants for water in sucrose, citric acid and α-pinene SOA are shown by the black line, green line and orange bars, respectively. The upper limit on the diffusion constant that can be determined from the optical tweezers approach is shown by the dotted blue line. Diffusion constants and viscosities for the protein bovine serum albumin (BSA) in aqueous solution are shown by the purple bars; those for ozone in aqueous-BSA are shown by the blue bars. The diffusion constant for pyrene in α-pinene SOA is shown by the pink square. The range of diffusion coefficients for ozone in shikimic acid aerosol is shown by the red bar, measured over the RH range 12 to 71% RH. (b) The effective vapour pressures for MA in ternary aqueous sucrose/MA aerosol as a function of viscosity. (c) The dependencies of the reactive uptake coefficient for ozone on aqueous MA/sucrose, and N_2_O_5_ and OH on aqueous citric acid aerosol are shown by the diamonds, circles and triangles, respectively.

We summarise the full range of reported aerosol measurements that have investigated the relationship between molecular diffusion and viscosity in Fig. 6(a). Only direct and independent measurements of diffusion constants and viscosities are shown. The sensitivity to molecular size is shown for the relationship between diffusion constant and viscosity estimated from the Stokes–Einstein equation, and all measurements are compared with this treatment. For both binary aqueous-sucrose and aqueous-citric acid droplets, we have measured the water activity dependence of the viscosities^22^ and the diffusion constants of water are also known independently,^23^,^52^ with parameterisations available for both. The diffusion constants for water show increasing divergence from the Stokes–Einstein prediction as the viscosity increases for the aqueous-sucrose system, deviating by more than two orders of magnitude even at an intermediate viscosity of 10^4^ Pa s.

For aqueous-citric acid droplets, the correspondence between the diffusion constant and viscosity appears unphysical. For this system, the viscosity is measured at discrete RHs and parameterised in terms of RH. The determination of the diffusion constants requires an accurate parameterisation of the thermodynamic relationship between mass fraction of solute and water activity; however, this relationship was based on measurements that were only available above a water activity of ∼0.78 (up to a mass fraction of solute of ∼0.65), leading to the possibility of very large errors under drier conditions.^52^ The limited range of water activities over which the thermodynamic relation is based on experimental data (and, thus, over which diffusion constants can be related accurately to water activity and therefore viscosity) is indicated by the solid line in Fig. 6(a). Outside this range (indicated by the dashed green line), uncertainties in the thermodynamic treatment may lead to large errors in the retrieval of diffusion constants and a seemingly unphysical dependence on viscosity. This is a crucial point the importance of which cannot be overstated if accurate diffusion constants are to be measured: to estimate diffusion constants of water, the thermodynamic relationship between water activity and mass fraction of solute must be known accurately. Based on the unphysical relationship between viscosity and diffusion for citric acid shown here, we suggest that this relationship is very rarely known with the accuracy required. This thermodynamic relationship can only be derived from aerosol measurements that themselves are very often limited by water transport kinetics.

Measurements of viscosity^19^ and water diffusion constants^13^ have been made for the water soluble fraction of SOA derived from the ozonolysis of α-pinene and their relationship is shown in Fig. 6(a). Water transport in SOA appears to deviate even more markedly from the Stokes–Einstein prediction for this system than for sucrose, even at the low viscosity of 1 Pa s where the disparity may be as much as 3 orders of magnitude. Such extreme failure of the Stokes–Einstein equation at such low viscosity should be noted and must be due to the different chemical functionalities and molecular weights present in the viscous matrix through which the water must diffuse. By contrast, in the same SOA matrix, Abramson *et al.* report a diffusion constant for pyrene of 2.5 × 10^–21^ m^2^ s^–1^ under dry conditions.^7^ If the Stokes–Einstein equation can be assumed to be valid for such a large molecule diffusing in the matrix, this corresponds to a viscosity of 10^8^ Pa s, similar in magnitude but lower than the lower limit of the viscosity reported by Renbaum-Wolff *et al.* of 5 × 10^8^ Pa s in the RH range 25–30% (not shown on the figure).^19^ This may suggest that the Stokes–Einstein equation may lead to an under-estimate of the diffusion constant even for pyrene (or an over-estimate of the viscosity). If the viscosity were infact 10^12^ Pa s (entirely consistent with the viscosity measurements of Renbaum-Wolff *et al.*), the diffusion constant estimate from the Stokes–Einstein equation would be ∼1 × 10^–24^ m^2^ s^–1^ assuming a molecular radius of 0.4 nm; the actual measured value is a factor of >2000 larger than this.

The lower limit of the diffusion constants for bovine serum albumin (BSA) in water shown in Fig. 6(a) show some degree of correspondence with the viscosity, although the error bars are quite large.^53^ Measurements of viscosity above 10^9^ Pa s at RHs below 80% for this system are not shown in this figure but show a similar level of correspondence, with the upper limit of the diffusion constant falling on the Stokes–Einstein prediction but with the lower limit some 4–5 orders of magnitude higher. The diffusion coefficients of the oxidant, in this case ozone in aqueous BSA, are also included, clearly demonstrating the importance of the molecular weight and size of the species in determining diffusion constants. The errors in the viscosities of aqueous BSA (not shown) span over two orders of magnitude below and two orders of magnitude above the value shown.

We show in Fig. 6(b) the effective vapour pressures measured for MA from the ternary droplets (data from Fig. 3 as well as from other droplet measurements) to explore the scaling of the volatility of MA with viscosity. At viscosities >100 Pa s, a regime where evaporation is sufficiently slowed below the gas diffusion limited flux that diffusion within the particle is entirely limiting, the effective vapour pressure scales inversely with viscosity, falling by two orders of magnitude with a two order of magnitude increase in viscosity. At lower viscosities, the effective vapour pressure tends to the pure component value. Assuming a Stokes–Einstein law scaling of diffusion constant could be applied, this would be equivalent to saying that the suppression in the kinetics of MA would be unresolvable as the diffusion constant of MA tends to 10^–14^ m^2^ s^–1^, and that the diffusion constant of MA decreases to a value of order 10^–15^ to 10^–17^ as the viscosity increases to a value of between 10^3^ and 10^5^ Pa s; however, some caution should be exercised in interpreting these numbers quantitatively.

When considering the relationship between viscosities and diffusion constants for oxidant molecules or other trace gas absorption, the available data are sparse (Fig. 6(c)). In particles of levoglucosan, with diameters in the range 120 to 267 nm, Slade *et al.* observed suppressions in the uptake coefficient of OH of a factor of 3 as the RH decreased from 40 to 10%, with the predicted diffusion constant of water showing a change of >10^6^ over this same range;^17^ no direct viscosity measurements are available for levoglucosan aerosol so we do not show the values in Fig. 6(c). A similar level of variation is seen in the uptake coefficients for ozone on MA/sucrose aerosol reported here over a viscosity range from 1 to 10^5^ Pa s. Uptake coefficients reported recently for N_2_O_5_ on citric acid aerosol show a marginally greater level of change with at most two orders of magnitude change with an increase in viscosity from 10^–1^ to 10^4^ Pa s.^54^ Uptake coefficients for OH on citric acid aerosol show a weak dependence on viscosity, with less than 1 order of magnitude variation over a viscosity range of more than 5 orders of magnitude.^55^ Similarly, the diffusion constant of ozone in BSA was estimated to vary over only ∼2 orders of magnitude even though the viscosity varied over 11 orders of magnitude.^53^ The diffusion constant for ozone in shikimic acid was inferred to decrease by more than 3 orders of magnitude as the RH was decreased from 71 to 12%, but no viscosity measurements are available for this system (Fig. 6(a)).^16^ Clearly suppression in oxidation kinetics (through uptake coefficients) or diffusion constants (for oxidants) do not seem to show the same level of sensitivity to viscosity as the diffusion constants of larger organic molecules with variations that may even be smaller than observed for water. This may reflect the greater strength of intermolecular interactions between water and the molecules forming the viscous matrices than between oxidants (such as OH and O_3_) and the matrix.

In summary, we have shown that the viscosity and diffusion constants of molecules in organic aerosol are strongly dependent on composition: although water clearly acts as a plasticiser, the presence of different organic moieties can lead to significant changes in the viscosity of the organic matrix. Largely two extremes have been considered thus far, simple binary mixtures of a single organic species and water, and complex mixtures (SOA). To retrieve accurate values for diffusion constants, the thermodynamic relationships that underpin such kinetic determinations, defining the initial and final state that the system must pass between, must be accurately known although such data are often not available. By contrast, the diffusion constants of similarly sized organic molecules within the matrix may be well represented by the Stokes–Einstein equation. Finally, the variation in uptake coefficients and diffusion constants for oxidants and small weakly interacting molecules may be much less dependent on viscosity than the diffusion constants of more strongly interacting molecules such as water; even for water, the Stokes–Einstein prediction of water transport is poor even at viscosities as low as 1 Pa s.

## Supplementary Material

Supplementary information
