# Supplementary material for: Diffusion and reactivity in ultraviscous aerosol and the correlation with particle viscosity
Source: Chem Sci. 2015 Nov 10;7(2):1298–308. doi: 10.1039/c5sc03223g (PMC5975831; doi:10.1039/c5sc03223g)
Supplement: Supplementary file 1 [file SC-007-C5SC03223G-s001.pdf]

Electronic Supplementary Material (ESI) for Chemical Science.
This journal is © The Royal Society of Chemistry 2015

Supplementary Information:

Diffusion Constants and Reactivity in Ultraviscous Aerosol and the Correlation with Particle Viscosity

Frances H. Marshall,[1] Rachael E.H. Miles, [1] Young-Chul Song, [1] Peter B. Ohm,[2]
Rory M. Power,[1,3] Jonathan P. Reid,[1]* and Cari S. Dutcher[2]

[1] *School of Chemistry, University of Bristol, Bristol, BS8 1TS, UK*

[2] *Department of Mechanical Engineering, University of Minnesota,*
111 Church Street SE, Minneapolis, MN 55455, USA

[3] *Max Planck Institute of Molecular Cell Biology and Genetics, Dresden, 01307, Germany*

Reactive Uptake Coefficients[1,2]

If it is assumed that all the reactive gas molecules in a flux react with the particle (with concentration [MA]), in which the uptake of O_3 is equal to the loss of MA, the rate of change in the concentration of MA can be expressed as:

$$\frac{d[MA]}{dt} = -\frac{3P_{O_3}\bar{c}\gamma}{4RTr} \qquad (1)$$

Where P_{O_3} is the partial pressure of ozone in the gas phase, $\bar{c}$ is the mean speed of MA in the gas phase, γ is the probability of an ozone molecule colliding with the particle, and r is the radius of the particle.[3] It is thought that the reaction between maleic acid and ozone exhibits two key limiting loss processes; loss of maleic acid via reaction with ozone in the bulk and loss via reaction with ozone at the particle surface, such that the net uptake of ozone is described by the sum of the uptake due to reaction at the surface (Γ_{surf}) and reaction in the bulk (Γ_{bulk}).

$$\gamma = \Gamma_{bulk} + \Gamma_{surf} \qquad (2)$$

Substituting this into equation 1, results in[4]

$$\frac{d[MA]}{dt} = -\frac{3P_{O_3}\bar{c}}{4RTr}(\Gamma_{bulk} + \Gamma_{surf}) \qquad (3)$$

As long as the concentration of ozone at the surface is not significantly affected by either process, it is possible to assume that the processes are decoupled from one another, allowing for γ to be solved for two limiting cases for which functional forms of Γ_{bulk} and Γ_{surf} have been determined. The two limits that apply are as follows:

1) $\Gamma_{surf} \ll \Gamma_{bulk}$ i.e. $\gamma = \Gamma_{bulk}$

2) $\Gamma_{surf} \gg \Gamma_{bulk}$ i.e. $\gamma = \Gamma_{surf}$

The heterogeneous oxidative ageing of aqueous MA has been shown to proceed via a bulk reaction limited by ozone diffusion, and as such ozone uptake due to reaction at the surface can be considered negligible.[4] This leads to the rate of change of ozone concentration within the particle that can be described by the differential:

$$\frac{\partial[O_3]}{dt} = D\nabla^2[O_3] - k[O_3] \qquad (4)$$

where $D\nabla^2[O_3]$ represents the diffusion of ozone in the particle, and $k[O_3]$ is the reactive loss. *D* is the diffusion coefficient for ozone in MA. $k = k_2[MA]$, the first order rate coefficient (s^{-1}) for a reaction between ozone and MA.

It is possible to solve the equation analytically if certain conditions are adhered to, such as assuming that the concentration of MA is uniform throughout the particle, the ozone concentration is in the steady state, and the particle is spherical and homogenous with constant radius r. On application of these, the steady state ozone concentration as a function of position within the particle can be assumed from which the flux of ozone into the particle can be calculated, and then normalised to the collision rate of ozone as described by Hanson and Lovejoy to yield[5]

$$\Gamma_{bulk} = \frac{flux_{surface}4RT}{P_{O_3}\bar{c}} \equiv \frac{4HRTD}{\bar{c}\quad l}\left(\coth\left({}^{r}/_{l}\right) - {}^{l}/_{r}\right) \qquad (5)$$

where *H* is the Henrys law solubility constant of ozone in MA (mol dm^{-3} atm^{-1}), and *l* is the diffuso-reactive length, the characteristic distance that an O_3 molecule typically diffuses before it reacts, equal to[4,5]

$$l = \sqrt{\frac{D_{O_3}}{k_2\,[MA]}} \qquad (6)$$

Substituting back into equation 3 yields:

$$\frac{d[MA]}{dt} = -\frac{3P_{O_3}HD_{O_3}}{r\quad l}\left(\coth\left({}^{r}/_{l}\right) - {}^{l}/_{r}\right) \qquad (7)$$

Although this equation cannot be solved analytically, it can be solved numerically assuming two limiting cases for l. Previous studies on the ozonolysis of aqueous maleic acid indicate that the reaction is limited by the diffusion of ozone into the particle bulk, however the increase in viscosity on addition of sucrose to the binary solution allows for the rate of the reaction to also be limited by slow kinetics within the particle bulk. As is the case, both scenarios for the diffuso-reactive length must be considered.

Case 1 – Slow kinetics within the particle bulk[1]

In this limit, coth(r/l) can be approximated by a Taylor expansion such that $\coth\left({}^{r}/_{l}\right) - {}^{l}/_{r} \cong \frac{r}{3l}$ lending to:

$$\frac{d[MA]}{dt} = - P_{O_3} H k_2 [MA] \qquad (8)$$

Solving this differential, assuming the boundary condition whereby at t=0 s, [MA] = $[MA]_0$, and assuming the identity $\frac{[MA]_t}{[MA]_0} = I_{v_{C-H}}$ (a measurable quantity in our experiments):

$$I_{v_{C-H}} = e^{-P_{O_3} H k_2 t} \qquad (9)$$

Rewriting this in terms of the uptake coefficient, γ, yields:

$$\gamma = \frac{4RTrHk_2[MA]}{3\bar{c}} \qquad (10)$$

Case 2 – Diffusion limited[2]

In a reaction limited by the diffusion of ozone and occurring near the surface, $\coth\left({}^{r}/_{l}\right) - {}^{l}/_{r}$ tends to asymptotic unity, such that

$$\frac{d[MA]}{dt} = - \frac{3P_{O_3} H \sqrt{D_{O_3} k_2 [MA]}}{r} \qquad (11)$$

Again, solving this differential, assuming the boundary condition whereby at t=0 s, [MA] = $[MA]_0$, and assuming the identity $\frac{[MA]_t}{[MA]_0} = I_{v_{C-H}}$ (a measurable quantity in our experiments):

$$I_{v_{C-H}} = 1 - \left(\frac{3P_{O_3} H \sqrt{D_{O_3} k_2}}{2r[MA]_0} t \right)^2 \qquad (12)$$

Rewriting this in terms of the uptake coefficient, γ, yields:

$$I_{v_{C-H}} = 1 - \left(\frac{3P_{O_3} \gamma_{MA} \bar{c}}{8RTr[MA]_0} t \right)^2 \qquad (13)$$

Table S1

RH / %	Error / %	Log (Viscosity / Pa s)	Log (Error / Pa s)	Mass Ratio MA:Sucrose
**Maleic Acid / Sucrose**				
14.7	0.3	5.15	0.11	0.12
15.4	0.3	4.78	0.49	0.09
21.1	0.4	3.93	-	0.09
23.2	0.5	4.43	0.36	0.12
30.6	0.6	2.47	0.25	0.11
33.0	0.7	3.83	0.57	0.14
35.9	0.7	2.96	-	0.10
42.4	0.9	2.53	0.44	0.09
46.5	0.9	2.08	-	0.11
52.8	1.1	2.59	0.75	0.12
55.8	1.1	1.88	-	0.10
62.8	1.3	1.96	0.23	0.13
42.3	0.8	6.18	-	0.02
52.3	1.0	4.46	-	0.04
**Citric Acid**				
12.4	0.2	2.72	2.63	-
12.4	0.2	3.50	3.35	-
15.4	0.3	3.12	2.63	-
15.6	0.3	2.82	2.63	-
16.9	0.3	3.80	3.35	-
18.6	0.4	3.58	3.02	-
19.7	0.4	2.92	2.38	-
20	0.4	3.37	3.02	-
22.9	0.5	2.69	2.38	-
26.1	0.5	2.38	3.06	-
26.7	0.5	3.34	3.06	-
28.5	0.6	2.24	3.06	-
30.4	0.6	2.19	2.38	-
33.4	0.7	1.57	1.21	-
38.3	0.8	1.14	1.21	-
42.4	0.8	0.22	-0.25	-
47.3	0.9	0.83	-	-
48.3	1.0	-0.34	-0.25	-
50.2	1.0	-0.01	-0.25	-
56.7	1.1	-0.32	-0.25	-
65.0	1.3	-0.36	-0.56	-

Table S2

System	Parameterisation	Error
Sucrose	$\log \eta = 1.01514 \times 10^{-3}(RH)^2 - 0.292865(RH) + 16.1351$	$0.10564 \times 10^{-3}(RH)^2 + 0.026529\,(RH) + 1.5965$
Sucrose/ MA	$\log \eta = -4.6071 \times 10^{-4}(RH)^2 - 3.6068 \times 10^{-2}(RH) + 5.2139$	$1.28763 \times 10^{-4}(RH)^2 + 0.033856\,(RH) + 2.09794$
Citric Acid	$\log \eta = 2.6421 \times 10^{-4}(RH)^2 - 0.1106(RH) + 5.4220$	-

References

1 J. J. Nájera, C. J. Percival and A. B. Horn, *Phys. Chem. Chem. Phys.* **12** (2010) 11417–11427.

2 M. D. King, K. C. Thompson, A. D. Ward, C. Pfrang and B. R. Hughes, *Faraday Discuss.* **137** **(**2008) 173–192.

3 D. R. Worsnop, J. W. Morris, Q. Shi, P. Davidovits, and C. E. Kolb, *Geophys. Res. Lett.* **29** (2002) 1996.

4 G. D. Smith, E. Woods, C. L. DeForest, T. Baer and R. E. Miller, *J. Phys. Chem. A* **106** (2002) 8085–8095

5 D. R. Hanson and E. R. Lovejoy, *Science*, **267** (1994)1326–1328.
